# Supplementary material for: Heparin-based hydrogel scaffolding alters the transcriptomic profile and increases the chemoresistance of MDA-MB-231 triple-negative breast cancer cells
Source: Biomater Sci. 2020 Feb 13;8(10):2786–96. doi: 10.1039/c9bm01481k (PMC7497406; doi:10.1039/c9bm01481k)
Supplement: Supplementary file 2 [file BM-008-C9BM01481K-s002.zip › Supplementary File 4/HP-BhydrogelvControl/Pathways/my_analysis.Gsea.1545201079907/HALLMARK_E2F_TARGETS.html]

Details for gene set HALLMARK\_E2F\_TARGETS[GSEA]

|  || Dataset | expr.class.cls#HYD\_versus\_CONTROL.class.cls#HYD\_versus\_CONTROL\_repos |
| Phenotype | class.cls#HYD\_versus\_CONTROL\_repos |
| Upregulated in class | CONTROL |
| GeneSet | HALLMARK\_E2F\_TARGETS |
| Enrichment Score (ES) | -0.34180838 |
| Normalized Enrichment Score (NES) | -1.6385235 |
| Nominal p-value | 0.0 |
| FDR q-value | 0.006701969 |
| FWER p-Value | 0.031 |
Table: GSEA Results Summary

  

Fig 1: Enrichment plot: HALLMARK\_E2F\_TARGETS      
 Profile of the Running ES Score & Positions of GeneSet Members on the Rank Ordered List

  

| PROBE | DESCRIPTION (from dataset) | GENE SYMBOL | GENE\_TITLE | RANK IN GENE LIST | RANK METRIC SCORE | RUNNING ES | CORE ENRICHMENT || 1 | PNN | na |  |  | 1859 | 1.422 | -0.0839 | No |
| 2 | WDR90 | na |  |  | 2715 | 1.112 | -0.1181 | No |
| 3 | ILF3 | na |  |  | 2804 | 1.084 | -0.1121 | No |
| 4 | RAD50 | na |  |  | 2975 | 1.043 | -0.1109 | No |
| 5 | CDC25B | na |  |  | 3153 | 0.999 | -0.1105 | No |
| 6 | DDX39A | na |  |  | 3637 | 0.885 | -0.1273 | No |
| 7 | MYC | na |  |  | 3771 | 0.854 | -0.1259 | No |
| 8 | POLD1 | na |  |  | 3872 | 0.839 | -0.1230 | No |
| 9 | POLE | na |  |  | 3906 | 0.832 | -0.1166 | No |
| 10 | LIG1 | na |  |  | 3913 | 0.830 | -0.1089 | No |
| 11 | POLA2 | na |  |  | 4005 | 0.811 | -0.1058 | No |
| 12 | DNMT1 | na |  |  | 4357 | 0.740 | -0.1170 | No |
| 13 | TIMELESS | na |  |  | 4544 | 0.703 | -0.1199 | No |
| 14 | WEE1 | na |  |  | 4603 | 0.692 | -0.1162 | No |
| 15 | MYBL2 | na |  |  | 4657 | 0.683 | -0.1124 | No |
| 16 | SMC1A | na |  |  | 4737 | 0.670 | -0.1100 | No |
| 17 | KIF18B | na |  |  | 4808 | 0.657 | -0.1073 | No |
| 18 | NOP56 | na |  |  | 4859 | 0.647 | -0.1036 | No |
| 19 | CIT | na |  |  | 4890 | 0.644 | -0.0989 | No |
| 20 | TRA2B | na |  |  | 4957 | 0.634 | -0.0962 | No |
| 21 | TACC3 | na |  |  | 5096 | 0.614 | -0.0975 | No |
| 22 | ESPL1 | na |  |  | 5108 | 0.612 | -0.0921 | No |
| 23 | KIF22 | na |  |  | 5147 | 0.606 | -0.0881 | No |
| 24 | CENPM | na |  |  | 5320 | 0.580 | -0.0915 | No |
| 25 | CDC25A | na |  |  | 5482 | 0.555 | -0.0946 | No |
| 26 | MCM7 | na |  |  | 5533 | 0.548 | -0.0919 | No |
| 27 | MCM2 | na |  |  | 5595 | 0.539 | -0.0898 | No |
| 28 | AURKB | na |  |  | 5662 | 0.530 | -0.0881 | No |
| 29 | TBRG4 | na |  |  | 6008 | 0.483 | -0.1016 | No |
| 30 | MXD3 | na |  |  | 6021 | 0.482 | -0.0975 | No |
| 31 | CDKN1B | na |  |  | 6043 | 0.481 | -0.0939 | No |
| 32 | CDKN2C | na |  |  | 6116 | 0.469 | -0.0932 | No |
| 33 | EZH2 | na |  |  | 6297 | 0.445 | -0.0983 | No |
| 34 | DONSON | na |  |  | 6370 | 0.434 | -0.0978 | No |
| 35 | PAN2 | na |  |  | 6582 | 0.405 | -0.1050 | No |
| 36 | LUC7L3 | na |  |  | 6708 | 0.391 | -0.1078 | No |
| 37 | PSMC3IP | na |  |  | 7055 | 0.353 | -0.1225 | No |
| 38 | TCF19 | na |  |  | 7093 | 0.348 | -0.1211 | No |
| 39 | HMGB2 | na |  |  | 7660 | 0.284 | -0.1481 | No |
| 40 | DSCC1 | na |  |  | 8141 | 0.241 | -0.1710 | No |
| 41 | UNG | na |  |  | 8156 | 0.239 | -0.1694 | No |
| 42 | SLBP | na |  |  | 8263 | 0.224 | -0.1728 | No |
| 43 | UBE2S | na |  |  | 8271 | 0.223 | -0.1710 | No |
| 44 | BARD1 | na |  |  | 8323 | 0.217 | -0.1715 | No |
| 45 | H2AFX | na |  |  | 8328 | 0.216 | -0.1696 | No |
| 46 | MCM5 | na |  |  | 8383 | 0.209 | -0.1705 | No |
| 47 | DIAPH3 | na |  |  | 8587 | 0.195 | -0.1792 | No |
| 48 | GINS4 | na |  |  | 8598 | 0.192 | -0.1779 | No |
| 49 | MCM4 | na |  |  | 8791 | 0.168 | -0.1864 | No |
| 50 | SHMT1 | na |  |  | 8825 | 0.163 | -0.1865 | No |
| 51 | GINS1 | na |  |  | 8843 | 0.160 | -0.1858 | No |
| 52 | RAD51C | na |  |  | 9123 | 0.137 | -0.1992 | No |
| 53 | NOLC1 | na |  |  | 9142 | 0.135 | -0.1988 | No |
| 54 | MLH1 | na |  |  | 9158 | 0.133 | -0.1983 | No |
| 55 | LMNB1 | na |  |  | 9190 | 0.127 | -0.1987 | No |
| 56 | CCNE1 | na |  |  | 9260 | 0.122 | -0.2011 | No |
| 57 | RANBP1 | na |  |  | 9418 | 0.103 | -0.2084 | No |
| 58 | POLD2 | na |  |  | 9616 | 0.087 | -0.2179 | No |
| 59 | CDC20 | na |  |  | 9617 | 0.086 | -0.2171 | No |
| 60 | SPC24 | na |  |  | 9625 | 0.085 | -0.2166 | No |
| 61 | MCM3 | na |  |  | 9637 | 0.083 | -0.2164 | No |
| 62 | SPAG5 | na |  |  | 9660 | 0.079 | -0.2168 | No |
| 63 | BRMS1L | na |  |  | 9695 | 0.076 | -0.2178 | No |
| 64 | NUP107 | na |  |  | 9751 | 0.071 | -0.2200 | No |
| 65 | AK2 | na |  |  | 9863 | 0.056 | -0.2253 | No |
| 66 | DCTPP1 | na |  |  | 9940 | 0.047 | -0.2288 | No |
| 67 | RPA1 | na |  |  | 9943 | 0.047 | -0.2285 | No |
| 68 | POLE4 | na |  |  | 9962 | 0.044 | -0.2290 | No |
| 69 | GINS3 | na |  |  | 10047 | 0.035 | -0.2331 | No |
| 70 | ATAD2 | na |  |  | 10358 | 0.011 | -0.2493 | No |
| 71 | NCAPD2 | na |  |  | 10385 | 0.008 | -0.2506 | No |
| 72 | TK1 | na |  |  | 10446 | 0.002 | -0.2537 | No |
| 73 | NAA38 | na |  |  | 10485 | -0.003 | -0.2557 | No |
| 74 | HELLS | na |  |  | 10515 | -0.007 | -0.2571 | No |
| 75 | TFRC | na |  |  | 10516 | -0.007 | -0.2571 | No |
| 76 | ASF1A | na |  |  | 10778 | -0.035 | -0.2705 | No |
| 77 | RFC2 | na |  |  | 10894 | -0.053 | -0.2760 | No |
| 78 | PLK1 | na |  |  | 10895 | -0.053 | -0.2755 | No |
| 79 | TUBG1 | na |  |  | 10996 | -0.065 | -0.2801 | No |
| 80 | STAG1 | na |  |  | 11103 | -0.080 | -0.2849 | No |
| 81 | POLD3 | na |  |  | 11139 | -0.085 | -0.2859 | No |
| 82 | SMC4 | na |  |  | 11306 | -0.090 | -0.2938 | No |
| 83 | MAD2L1 | na |  |  | 11353 | -0.097 | -0.2953 | No |
| 84 | KIF4A | na |  |  | 11399 | -0.101 | -0.2966 | No |
| 85 | CDCA8 | na |  |  | 11406 | -0.102 | -0.2960 | No |
| 86 | SRSF1 | na |  |  | 11410 | -0.102 | -0.2951 | No |
| 87 | NME1 | na |  |  | 11542 | -0.117 | -0.3009 | No |
| 88 | CDKN3 | na |  |  | 11708 | -0.139 | -0.3082 | No |
| 89 | MCM6 | na |  |  | 11728 | -0.142 | -0.3078 | No |
| 90 | CDKN1A | na |  |  | 11746 | -0.145 | -0.3073 | No |
| 91 | PRKDC | na |  |  | 11751 | -0.145 | -0.3061 | No |
| 92 | PA2G4 | na |  |  | 11816 | -0.152 | -0.3080 | No |
| 93 | KIF2C | na |  |  | 11924 | -0.166 | -0.3120 | No |
| 94 | SNRPB | na |  |  | 12003 | -0.172 | -0.3144 | No |
| 95 | DEK | na |  |  | 12016 | -0.175 | -0.3133 | No |
| 96 | SMC6 | na |  |  | 12051 | -0.180 | -0.3134 | No |
| 97 | BRCA1 | na |  |  | 12249 | -0.206 | -0.3217 | No |
| 98 | CDCA3 | na |  |  | 12268 | -0.209 | -0.3206 | No |
| 99 | CHEK2 | na |  |  | 12299 | -0.213 | -0.3201 | No |
| 100 | ORC6 | na |  |  | 12622 | -0.237 | -0.3348 | No |
| 101 | SUV39H1 | na |  |  | 12643 | -0.239 | -0.3335 | No |
| 102 | ANP32E | na |  |  | 12802 | -0.258 | -0.3393 | Yes |
| 103 | PRIM2 | na |  |  | 12834 | -0.262 | -0.3384 | Yes |
| 104 | UBE2T | na |  |  | 12862 | -0.265 | -0.3372 | Yes |
| 105 | ING3 | na |  |  | 12917 | -0.273 | -0.3374 | Yes |
| 106 | PMS2 | na |  |  | 12931 | -0.275 | -0.3354 | Yes |
| 107 | RFC3 | na |  |  | 13005 | -0.284 | -0.3365 | Yes |
| 108 | NUP153 | na |  |  | 13020 | -0.287 | -0.3344 | Yes |
| 109 | PHF5A | na |  |  | 13067 | -0.293 | -0.3340 | Yes |
| 110 | SSRP1 | na |  |  | 13070 | -0.294 | -0.3312 | Yes |
| 111 | RRM2 | na |  |  | 13076 | -0.296 | -0.3286 | Yes |
| 112 | LYAR | na |  |  | 13114 | -0.301 | -0.3276 | Yes |
| 113 | ORC2 | na |  |  | 13360 | -0.330 | -0.3372 | Yes |
| 114 | ASF1B | na |  |  | 13428 | -0.342 | -0.3374 | Yes |
| 115 | SPC25 | na |  |  | 13457 | -0.346 | -0.3355 | Yes |
| 116 | RACGAP1 | na |  |  | 13570 | -0.365 | -0.3379 | Yes |
| 117 | TRIP13 | na |  |  | 13583 | -0.367 | -0.3349 | Yes |
| 118 | DLGAP5 | na |  |  | 13683 | -0.383 | -0.3364 | Yes |
| 119 | RAN | na |  |  | 13684 | -0.383 | -0.3327 | Yes |
| 120 | MSH2 | na |  |  | 13742 | -0.394 | -0.3318 | Yes |
| 121 | CTCF | na |  |  | 13744 | -0.395 | -0.3280 | Yes |
| 122 | PLK4 | na |  |  | 13826 | -0.410 | -0.3283 | Yes |
| 123 | CHEK1 | na |  |  | 13855 | -0.412 | -0.3258 | Yes |
| 124 | MELK | na |  |  | 13874 | -0.415 | -0.3227 | Yes |
| 125 | NASP | na |  |  | 14013 | -0.418 | -0.3258 | Yes |
| 126 | CCP110 | na |  |  | 14173 | -0.437 | -0.3299 | Yes |
| 127 | PRPS1 | na |  |  | 14176 | -0.438 | -0.3258 | Yes |
| 128 | LBR | na |  |  | 14304 | -0.464 | -0.3279 | Yes |
| 129 | BRCA2 | na |  |  | 14335 | -0.469 | -0.3249 | Yes |
| 130 | EED | na |  |  | 14401 | -0.477 | -0.3237 | Yes |
| 131 | EXOSC8 | na |  |  | 14431 | -0.480 | -0.3206 | Yes |
| 132 | AURKA | na |  |  | 14577 | -0.502 | -0.3233 | Yes |
| 133 | NBN | na |  |  | 14611 | -0.508 | -0.3201 | Yes |
| 134 | BIRC5 | na |  |  | 14629 | -0.512 | -0.3160 | Yes |
| 135 | USP1 | na |  |  | 14776 | -0.533 | -0.3185 | Yes |
| 136 | PPM1D | na |  |  | 14788 | -0.536 | -0.3138 | Yes |
| 137 | TMPO | na |  |  | 14804 | -0.538 | -0.3094 | Yes |
| 138 | TIPIN | na |  |  | 14806 | -0.539 | -0.3042 | Yes |
| 139 | RAD51AP1 | na |  |  | 14850 | -0.544 | -0.3011 | Yes |
| 140 | RAD1 | na |  |  | 14958 | -0.556 | -0.3013 | Yes |
| 141 | GSPT1 | na |  |  | 15339 | -0.624 | -0.3152 | Yes |
| 142 | PPP1R8 | na |  |  | 15358 | -0.627 | -0.3101 | Yes |
| 143 | STMN1 | na |  |  | 15496 | -0.649 | -0.3110 | Yes |
| 144 | H2AFZ | na |  |  | 15730 | -0.688 | -0.3165 | Yes |
| 145 | DEPDC1 | na |  |  | 15735 | -0.688 | -0.3100 | Yes |
| 146 | DCLRE1B | na |  |  | 15823 | -0.708 | -0.3077 | Yes |
| 147 | CENPE | na |  |  | 15857 | -0.715 | -0.3024 | Yes |
| 148 | NUDT21 | na |  |  | 15890 | -0.721 | -0.2971 | Yes |
| 149 | PCNA | na |  |  | 15928 | -0.728 | -0.2919 | Yes |
| 150 | XRCC6 | na |  |  | 15966 | -0.737 | -0.2867 | Yes |
| 151 | TOP2A | na |  |  | 15987 | -0.741 | -0.2805 | Yes |
| 152 | EIF2S1 | na |  |  | 16087 | -0.767 | -0.2783 | Yes |
| 153 | PAICS | na |  |  | 16135 | -0.775 | -0.2732 | Yes |
| 154 | CSE1L | na |  |  | 16145 | -0.776 | -0.2661 | Yes |
| 155 | RPA3 | na |  |  | 16182 | -0.784 | -0.2603 | Yes |
| 156 | RAD21 | na |  |  | 16290 | -0.809 | -0.2581 | Yes |
| 157 | NUP205 | na |  |  | 16374 | -0.827 | -0.2544 | Yes |
| 158 | MMS22L | na |  |  | 16395 | -0.831 | -0.2473 | Yes |
| 159 | HMMR | na |  |  | 16468 | -0.845 | -0.2429 | Yes |
| 160 | SRSF2 | na |  |  | 16515 | -0.858 | -0.2370 | Yes |
| 161 | PDS5B | na |  |  | 16521 | -0.859 | -0.2288 | Yes |
| 162 | E2F8 | na |  |  | 16667 | -0.890 | -0.2278 | Yes |
| 163 | SMC3 | na |  |  | 16746 | -0.913 | -0.2230 | Yes |
| 164 | TP53 | na |  |  | 16860 | -0.944 | -0.2197 | Yes |
| 165 | CDK1 | na |  |  | 16949 | -0.970 | -0.2149 | Yes |
| 166 | CDK4 | na |  |  | 16963 | -0.974 | -0.2061 | Yes |
| 167 | XPO1 | na |  |  | 16967 | -0.975 | -0.1967 | Yes |
| 168 | MTHFD2 | na |  |  | 16986 | -0.982 | -0.1881 | Yes |
| 169 | CCNB2 | na |  |  | 17048 | -0.999 | -0.1816 | Yes |
| 170 | POP7 | na |  |  | 17058 | -1.004 | -0.1723 | Yes |
| 171 | CKS1B | na |  |  | 17139 | -1.027 | -0.1665 | Yes |
| 172 | PSIP1 | na |  |  | 17173 | -1.039 | -0.1581 | Yes |
| 173 | RNASEH2A | na |  |  | 17184 | -1.044 | -0.1484 | Yes |
| 174 | UBR7 | na |  |  | 17249 | -1.066 | -0.1414 | Yes |
| 175 | KPNA2 | na |  |  | 17340 | -1.098 | -0.1354 | Yes |
| 176 | PTTG1 | na |  |  | 17418 | -1.121 | -0.1286 | Yes |
| 177 | HMGB3 | na |  |  | 17427 | -1.125 | -0.1180 | Yes |
| 178 | DUT | na |  |  | 17602 | -1.198 | -0.1155 | Yes |
| 179 | SYNCRIP | na |  |  | 17710 | -1.247 | -0.1090 | Yes |
| 180 | BUB1B | na |  |  | 17744 | -1.259 | -0.0984 | Yes |
| 181 | NAP1L1 | na |  |  | 17791 | -1.282 | -0.0883 | Yes |
| 182 | DCK | na |  |  | 17810 | -1.291 | -0.0767 | Yes |
| 183 | PRDX4 | na |  |  | 17878 | -1.323 | -0.0673 | Yes |
| 184 | RBBP7 | na |  |  | 17886 | -1.326 | -0.0548 | Yes |
| 185 | RFC1 | na |  |  | 17908 | -1.336 | -0.0428 | Yes |
| 186 | HNRNPD | na |  |  | 18008 | -1.388 | -0.0345 | Yes |
| 187 | ZW10 | na |  |  | 18065 | -1.424 | -0.0236 | Yes |
| 188 | RPA2 | na |  |  | 18162 | -1.475 | -0.0142 | Yes |
| 189 | HUS1 | na |  |  | 18375 | -1.622 | -0.0096 | Yes |
| 190 | CKS2 | na |  |  | 18444 | -1.669 | 0.0031 | Yes |
| 191 | CBX5 | na |  |  | 18643 | -1.839 | 0.0106 | Yes |
| 192 | IPO7 | na |  |  | 18708 | -1.900 | 0.0258 | Yes |
Table: GSEA details [plain text format]

  

Fig 2: HALLMARK\_E2F\_TARGETS      
 Blue-Pink O' Gram in the Space of the Analyzed GeneSet

  

Fig 3: HALLMARK\_E2F\_TARGETS: Random ES distribution      
 Gene set null distribution of ES for **HALLMARK\_E2F\_TARGETS**
